# Cranial irradiation induces bone marrow-derived microglia in adult mouse brain tissue

**DOI:** 10.1093/jrr/rru015

**Published:** 2014-04-04

**Authors:** Noriyuki Okonogi, Kazuhiro Nakamura, Yoshiyuki Suzuki, Nana Suto, Kazutomo Suzue, Takuya Kaminuma, Takashi Nakano, Hirokazu Hirai

**Affiliations:** 1 Department of Radiation Oncology, Gunma University Graduate School of Medicine, 3-39-22 Showa-machi, Maebashi, Gunma 371-8511, Japan; 2 Department of Neurophysiology, Gunma University Graduate School of Medicine, 3-39-22 Showa-machi, Maebashi, Gunma 371-8511, Japan; 3 Department of Parasitology, Gunma University Graduate School of Medicine, 3-39-22 Showa-machi, Maebashi, Gunma 371-8511, Japan

**Keywords:** microglia, bone marrow-derived microglia, cranial irradiation, transgenic mice

## Abstract

Postnatal hematopoietic progenitor cells do not contribute to microglial homeostasis in adult mice under normal conditions. However, previous studies using whole-body irradiation and bone marrow (BM) transplantation models have shown that adult BM cells migrate into the brain tissue and differentiate into microglia (BM-derived microglia; BMDM). Here, we investigated whether cranial irradiation alone was sufficient to induce the generation of BMDM in the adult mouse brain. Transgenic mice that express green fluorescent protein (GFP) under the control of a murine stem cell virus (MSCV) promoter (MSCV-GFP mice) were used. MSCV-GFP mice express GFP in BM cells but not in the resident microglia in the brain. Therefore, these mice allowed us to detect BM-derived cells in the brain without BM reconstitution. MSCV–GFP mice, aged 8–12 weeks, received 13.0 Gy irradiation only to the cranium, and BM-derived cells in the brain were quantified at 3 and 8 weeks after irradiation. No BM-derived cells were detected in control non-irradiated MSCV-GFP mouse brains, but numerous GFP-labeled BM-derived cells were present in the brain stem, basal ganglia and cerebral cortex of the irradiated MSCV-GFP mice. These BM-derived cells were positive for Iba1, a marker for microglia, indicating that GFP-positive BM-derived cells were microglial in nature. The population of BMDM was significantly greater at 8 weeks post-irradiation than at 3 weeks post-irradiation in all brain regions examined. Our results clearly show that cranial irradiation alone is sufficient to induce the generation of BMDM in the adult mouse.

## INTRODUCTION

Microglia are immune effector cells of the central nervous system (CNS) and play an important role in processes such as phagocytosis [1] and antigen-presentation [2], and in defense against the neurodegeneration of neural tissue [3]. In the prenatal state, hematopoietic progenitor cells differentiate into microglia. However, under normal conditions in the postnatal state, hematopoietic progenitor cells do not contribute to microglial homeostasis in the adult mouse [4]. However, recent studies have shown that adult bone marrow (BM) cells may have the potential to migrate to the brain tissue and differentiate into microglia under non-normal conditions [5, 6]. Microglia that have migrated from the BM to the adult brain tissue have been termed BM-derived microglia (BMDM).

The combination of BM transplantation with whole-body irradiation is one specific condition that induces BMDM formation. Priller *et al.* have reported that sublethal whole-body irradiation given as a pretreatment to BM transplantation induced the migration of BM cells to the adult mouse brain [5]. They concluded that BM transplantation, rather than irradiation, primarily contributes to the induction of BM cells seen in the brain tissue. In a different study, Burrell *et al.* showed that irradiation alone would induce the migration of BM cells into the brain [6]. In Burrell's study, sublethal irradiation to the whole-body except for the cranial region was given prior to BM transplantation. The mice then received cranial irradiation after BM reconstitution. This resulted in BM cell migration to the irradiated brain region. The authors further showed that the majority of migrated BM cells had differentiated into microglia. They concluded that irradiation alone induced the migration of BM cells into the brain. However, the experiment by Burrell *et al.* included both BM transplantation and sublethal whole-body irradiation. Therefore, it remains unclear whether irradiation, specifically, cranial irradiation, alone is sufficient to induce BMDM generation or if co-treatment with BM transplantation is required.

In the present study, transgenic mice expressing green fluorescent protein (GFP) in the BM cells, but not in brain-resident microglia, were used. We examined whether cranial irradiation is sufficient to induce BMDM in the adult mouse brain. We further determined the brain regions preferentially targeted during migration and the temporal change in BMDM number following cranial irradiation.

## MATERIALS AND METHODS

### Animals

**Fig. 1.**
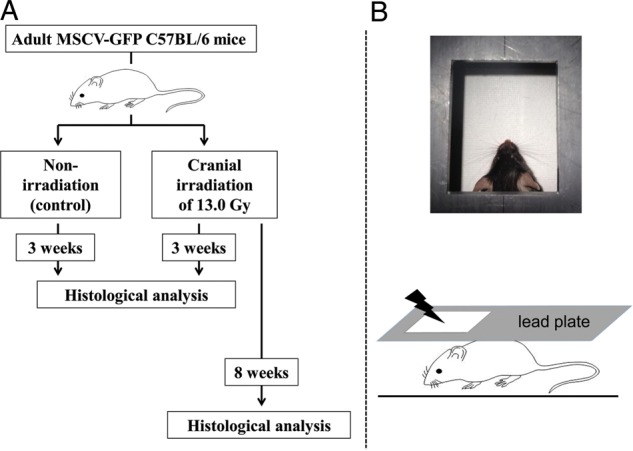
Experimental design. (**A**) Schematic depicting the experimental protocol. Eight MSCV-GFP mice received 13 Gy cranial irradiation under anesthesia. Four MSCV-GFP mice that received anesthesia but were not subjected to irradiation were used as controls. Four irradiated and four non-irradiated mice were sacrificed for histological analysis 3 weeks post-treatment. The remaining four irradiated mice were sacrificed and histologically examined at 8 weeks post-irradiation. (**B**) For head-specific irradiation, a mouse was placed in the prone position, and the body except for the cranial region was shielded with lead plates.

All experiments were conducted in accordance with the guidelines of the Animal Care and Experimentation Committee of the Gunma University. The experimental design is shown in Fig. 1A. We used only male transgenic mice at the age of 8–12 weeks with a C57BL/6 background, which expressed GFP under the control of murine stem cell virus (MSCV) promoter (MSCV-GFP mice) [7]. Mice were subjected to cranial irradiation. MSCV–GFP mice express GFP in BM cells and cerebellar Purkinje cells but not in resident microglia [8], which enabled us to detect endogenous BM cells that migrate into the brain tissue without BM reconstitution.

### Cranial irradiation

Cranial irradiation was performed using a TITAN 225-S X-ray machine (Shimadzu Co., Tokyo, Japan) that generated 200 kV X-rays at a dose rate of 1.3 Gy/min. Mice were anesthetized with an intraperitoneal injection of pentobarbital (40 mg/kg), and were placed in a prone position. A lead plate (6 mm thick) containing a rectangular window was positioned over the mouse to shield the majority of the body (Fig. 1B) but allow exposure to just the head of the mouse through the window. This resulted in cranial region-specific irradiation. A total of 13 Gy of cranial irradiation was applied to the dorsal side of each mouse. MSCV–GFP mice aged 8–12 weeks received cranial irradiation (*n* = 8). Animals that underwent the same anesthesia protocol but did not receive irradiation served as a control group (*n* = 4) (Fig. 1).

### Immunofluorescent staining

Irradiated MSCV–GFP mice were sacrificed at 3 weeks (*n* = 4) or 8 weeks (*n* = 4) after treatment for histological analysis. Control MSCV-GFP mice that received only anesthesia were similarly examined at 3 weeks (*n* = 4) after the treatment.

Transcardial perfusion with 4% paraformaldehyde in phosphate-buffered saline (PBS) was performed prior to brain dissection. Dissected brain tissue was fixed in the same fixative solution for an additional 24 h. On the next day, the brains were washed in PBS and cut into 50-μm sagittal sections using a vibratome. The sections were double immunostained with rat monoclonal anti-GFP (1:1000; 04404-84; Nacalai Tesque, Kyoto, Japan) and rabbit polyclonal anti-Iba1 (1:1000; 019-19741; Wako, Osaka, Japan) antibodies, as described previously [9]. In brief, sections were incubated in the presence of 1 µg/ml anti-Iba1 and 1 µg/ml anti-GFP antibodies overnight. After three PBS washes, sections were incubated with the fluorescent secondary antibodies, Alexa Fluor 488 donkey anti-rat IgG (1:1000; Life Technologies, Gaithersburg, MD, USA) and Alexa Fluor 568 donkey anti-rabbit IgG (1:1000; Life Technologies) in PBS for 1 h at room temperature. The stained sections were mounted on glass slides after three PBS washes. We have confirmed that the immunoreactivity was specific by conducting negative control experiments that included secondary antibodies, but not primary antibodies.

### Quantification of resident microglia and bone marrow-derived microglia

**Fig. 2.**
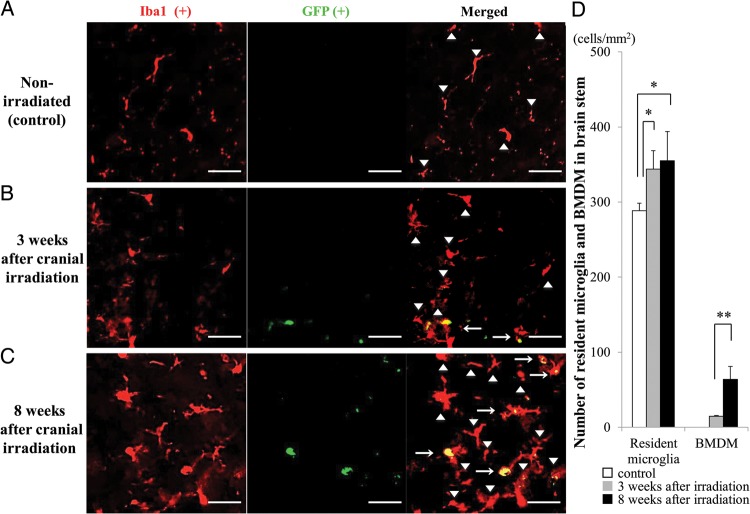
Induction of BMDM in the brain tissue following the cranial irradiation. (A–C) Representative images of the brain stem double immunolabeled for GFP and Iba1 from non-irradiated control mice (**A**) and irradiated mice at 3 (**B**) and 8 (**C**) weeks post-irradiation. Iba1-positive and GFP-negative resident microglial cells were observed in all groups (arrowheads), whereas Iba1- and GFP- double positive cells were present only in the irradiated groups (arrows). Scale bars, 40 µm. (**D**) Summarized graph showing the number of resident microglia (left columns) and BMDM (right columns) in 1 mm^2^ area of the brain stem. White bar: control group, gray bars: 3 weeks post-irradiation, black bars: 8 weeks post-irradiation. *P < 0.05, **P < 0.01.

**Fig. 3.**
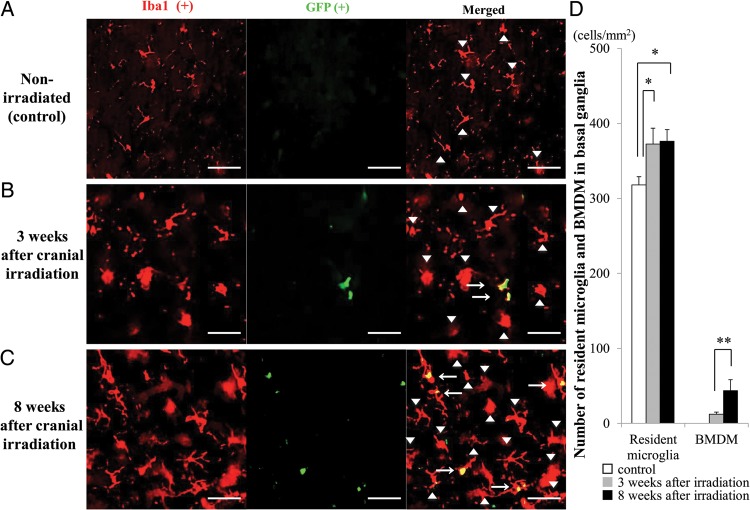
Migration of BMDM in the basal ganglia after the cranial irradiation. (A–C) Representative images of the basal ganglia double immunolabeled for GFP and Iba1 from non-irradiated control mice (**A**) and irradiated mice at 3 (**B**) and 8 (**C**) weeks post-irradiation. Similar to the brain stem, Iba1-positive and GFP-negative resident microglial cells were observed in all groups (arrowheads), and Iba1- and GFP-double-positive cells were present only in the irradiated groups (arrows). Scale bars, 40 µm. (**D**) Summarized graph showing the number of resident microglia (left columns) and BMDM (right columns) in 1 mm^2^ area of the basal ganglia. White bar: control group, gray bars: 3 weeks post-irradiation, black bars: 8 weeks post-irradiation. *P < 0.05, **P < 0.01.

**Fig. 4.**
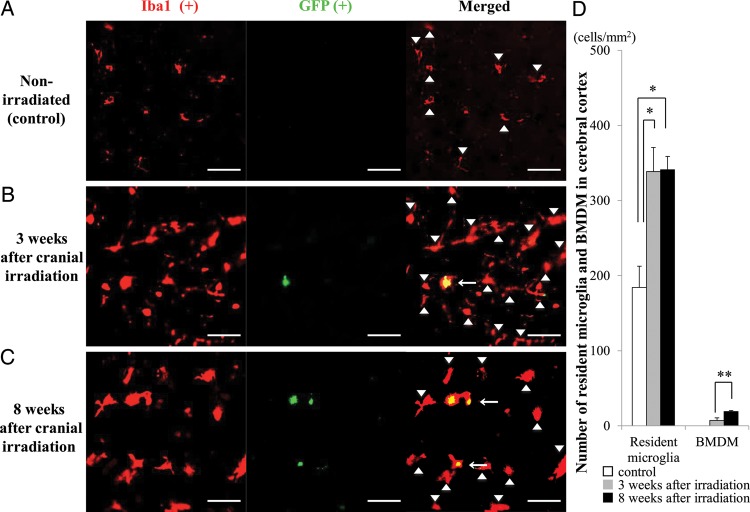
Migration of BMDM in the cerebral cortex after the cranial irradiation. (A–C) Representative images of the cerebral cortex double immunolabeled for GFP and Iba1 from non-irradiated control mice (**A**) and irradiated mice at 3 (**B**) and 8 (**C**) weeks post-irradiation. Similar to other regions, Iba1-positive and GFP-negative resident microglial cells were observed in all groups (arrowheads), and Iba1- and GFP-double-positive cells were present only in the irradiated groups (arrows). Scale bars, 40 µm. (**D**) Summarized graph showing the number of resident microglia (left columns) and BMDM (right columns) in 1 mm^2^ area of the cerebral cortex. White bar: control group, gray bars: 3 weeks post-irradiation, black bars: 8 weeks post-irradiation. *P < 0.05, **P < 0.01.

All sections were observed by the blind test method. All fluorescent images were obtained using a fluorescence microscope (BZ-9000; Keyence, Osaka, Japan) equipped with a ×20 objective lens. We counted the number of cells in five randomly selected regions/slice. In each focused brain region, five to six slices from four animals/group were used for the analysis. Sections were excited at 488 nm and 543 nm. All fluorescent images were analyzed with the BZ-II analysis program (Keyence). Both GFP- and Iba1-positive cells were automatically counted (Figs 2–4) and were further confirmed by visual inspection. In brief, the contrast of all obtained images was adjusted to the same condition (shadows: 60, Highlight: 150, γ-value: 1.0) in BZ-II analysis program (Keyence). Then, Keyence's ‘cell separation method’ was used to count individual cells. Cells <10 µm^2^ were excluded from counting. Microglia were immunolabeled for Iba1. Iba1-positive cells that were co-immunostained for GFP were derived from the BM and were therefore considered to be BMDM. The numbers of Iba1-positive and GFP-negative cells and Iba1- and GFP-double positive cells in 1 mm^2^ were counted.

### Statistical analysis

Significant differences were evaluated using ANOVA with a *post hoc* test and an unpaired two-tailed Student's *t*-test. Data were analyzed using group means with error bars reported as standard deviations (SDs). Differences were considered significant when the *P*-values were <0.05. All statistical analyses were performed using SPSS 16.0 for Mac (SPSS, Chicago, IL, USA).

## RESULTS

### Migration of BMDM into the brain tissue

Fig. 2A − C shows representative fluorescence images of the brain stem double immunostained for Iba1 and GFP from non-irradiated MSCV-GFP mice (Fig. 2A) and from irradiated MSCV-GFP mice at 3 (Fig. 2B) and 8 (Fig. 2C) weeks after cranial irradiation. Iba1-positive microglia were widely distributed in all groups. Quantitative data analysis showed that the number of resident microglia, which were defined as Iba1-positive and GFP-negative cells (arrowheads in Fig. 2A–C), was significantly higher at 3 (*P* < 0.05) and 8 (*P* < 0.05) weeks post-irradiation compared with non-irradiated controls (Fig. 2D). Notably, Iba1- and GFP-double positive cells (arrows in Fig. 2B and C), which were considered to be BMDM (hereafter referred to as BMDM), were observed only in the irradiated groups. Moreover, the density of BMDM was significantly higher at 8 weeks post-irradiation than at 3 weeks post-irradiation (*P* < 0.01, Fig. 2D).

Similar patterns were found in the basal ganglia (Fig. 3A − C) and cerebral cortex (Fig. 4A − C). The number of resident microglia in these regions was significantly higher in irradiated mice at both 3 (*P* < 0.05, each) and 8 weeks post-irradiation (*P* < 0.05, each), compared with the control, non-irradiated mice (Figs 3D and 4D). Similar to what was observed in the brain stem, Iba1- and GFP-double positive BMDM were also observed in these regions in the irradiated groups but not in the non-irradiated group.

### Comparison of the number of resident microglia and BMDM in different brain regions

**Fig. 5.**
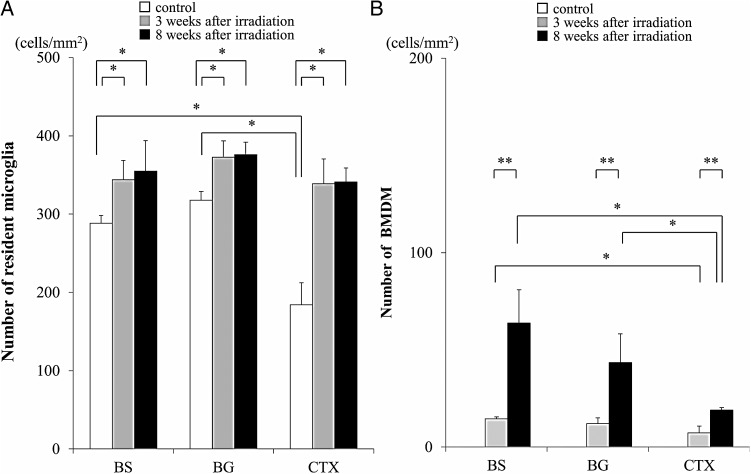
Regional differences in the number of resident microglia and BMDM. (A, B) Comparison of the number of resident microglia (**A**) and BMDM (**B**) in the brain stem (BS), basal ganglia (BG), and cerebral cortex (CTX) between control, non-irradiated mice (white columns) and irradiated mice at 3 (gray columns) or 8 (black columns) weeks post-irradiation. All values are presented as the mean ± SD, *P < 0.05, **P < 0.01.

Figure 5A and B show a time-course for the number of resident microglia and BMDM observed in the brain stem, basal ganglia, and cerebral cortex after cranial irradiation. Resident microglia density was almost comparable in the brain stem (290 ± 10 cells/mm^2^) and the basal ganglia (318 ± 11 cells/mm^2^) of control, non-irradiated mice, whereas it was significantly less in the cerebral cortex (184 ± 28 cells/mm^2^). As described above, cranial irradiation significantly increased resident microglia density in all three regions to 339–373 cells/mm^2^ at 3 weeks post-irradiation. No statistically significant differences in number were present between the groups (Fig. 5A). The resident microglia density in these three regions at 8 weeks after irradiation ranged from 341–376 cells/mm^2^, showing no further increase in the resident microglia population (Fig. 5A).

In contrast, the number of BMDM increased in a manner dependent on the time following irradiation. The number of BMDM at 3 weeks post-irradiation was highest in the brain stem (15 ± 1 cells/mm^2^), followed by the basal ganglia (12 ± 3 cells/mm^2^) and cerebral cortex (7 ± 3 cells/mm^2^). The number of BMDM in the cerebral cortex was significantly less than that in the brain stem (Fig. 5B). The difference between BMDM numbers in the brain regions expanded at 8 weeks post-irradiation. The number of BMDM at 8 weeks post-irradiation was 64 ± 17 cells/mm^2^ in the brain stem, 44 ± 15 cells/mm^2^ in the basal ganglia, and 19 ± 1 cells/mm^2^ in the cerebral cortex. Thus, the number of BMDM in each region increased 2.7–4.3 times during the period between 3 weeks and 8 weeks post-irradiation (Fig. 5B). At 8 weeks post-irradiation, the BMDM density in the brain stem was 1.5 times and 3.3 times higher than that observed in the basal ganglia and cerebral cortex, respectively. There were statistically significant differences in BMDM density between the brain stem or basal ganglia and cerebral cortex at 8 weeks post-irradiation (Fig. 5B). Thus, following cranial irradiation, BMDM were induced most densely in the brain stem, followed by the basal ganglia and cerebral cortex during our 8-week observation period.

## DISCUSSION

Although hematopoietic progenitor cells differentiate into microglia in the prenatal state [4], whether this occurs in the adult brain remains an unanswered question.

Priller *et al.* investigated whether microglia originated from hematopoietic cells during transient focal cerebral ischemia in mice that received transplantation of GFP-labeled BM cells after sublethal irradiation [5]. They showed that a massive infiltration of GFP-labeled round-shaped cells occurred in the ischemic cortex, striatum and hippocampus 24 h after transient middle cerebral artery occlusion. Donor BM-derived cells that had infiltrated the wounded parenchyma were shown to differentiate into Iba1-positive microglia. The authors also evaluated CNS microglial engraftment in a more selective lesion model, namely, transection of the fimbria-fornix. They obtained essentially the same result with this model [5]. These results reveal an enhanced microglial engraftment following CNS injury, which likely suggests mechanical destruction of the blood–brain barrier (BBB) as a promoting factor for the migration of BM cells into the brain. On the other hand, Priller *et al.* also applied unilateral facial nerve axotomy, which leaves the BBB intact, and ramified GFP-expressing cells were found to ensheath the axotomized motoneurons in the facial nucleus [5]. Collectively, they proposed that neurons might signal damage to circulating cells via specific molecular mediators, such as monocyte chemoattractant protein-1, as a mechanism by which BMDM is induced in the brain.

Although there are several studies showing the presence of BMDM in the adult brain [5, 6, 10, 11], it remains unclear whether BM transplantation, irradiation or both induce BMDM migration into the brain tissue because previous studies used BM transplantation to selectively label BM cells with GFP along with sublethal whole-body irradiation pretreatment. Sublethal whole body irradiation could cause inflammatory changes in many organs and tissues [12], leading to the release of various cytokines [13, 14]. Cytokines may subsequently affect the transplanted BM cells, potentially contributing to the induction of BMDM in the brain. Alternatively, allogeneic BM cells may trigger immune responses in the brain tissue, where they, in turn, receive signals driving their differentiation into BMDM [15, 16]. Thus, it remains unsolved whether cranial irradiation alone has the potential to induce BMDM in the brain.

In the present investigation, we addressed this question using MSCV-GFP mice that selectively express GFP in BM cells but not in the brain [8], with the exception of cerebellar Purkinje cells [8]. This expression did not interfere with the detection of BM-derived microglia. Therefore, the use of MSCV-GFP mice allowed us to examine the influence of cranial irradiation on BMDM induction without BM transplantation. Our results showed that the cranial irradiation of MSCV-GFP mice induced migration of endogenous (GFP-labeled) BM cells into numerous regions in the brain (in particular, the brain stem, basal ganglia, and cerebral cortex). The migrated BM cells were immunolabeled for Iba1, indicating that they were microglia in nature. BM cells entered the brain presumably due to the destruction of the BBB, and the BM cells differentiated into microglia, most likely via molecular mediators produced following brain injury. Although the mechanism of BMDM induction remains unclear, the present study clearly shows that cranial irradiation alone is sufficient to induce BMDM.

Burrell *et al.* studied the time-course of BMDM migration in the brain tissue from 1 d to 3 weeks after cranial irradiation in mice treated with both BM reconstitution and irradiation. The authors found that the number of BMDM increased over time until 3 weeks [6]. Our results showed that BMDM continued to increase in number up to 8 weeks post-irradiation, and at this time, the BMDM density was significantly higher than that observed at 3 weeks in the three brain regions examined. In contrast, there was no statistically significant difference in resident microglia density in the three brain regions between 3 weeks and 8 weeks after cranial irradiation (Fig. 5). These observations suggest that resident microglia migrate to target sites within 3 weeks after irradiation, whereas migration of BMDM to the brain tissue occurs continuously over a longer period. Priller's previous study [5] showed that migration of BMDM to the brain tissue continued to increase for up to 15 weeks. In this context, it is possible that resident microglia are induced into the injured sites relatively quickly, and then BMDM migrate gradually to the same sites over a longer time-span.

Regarding the relationship between apoptosis and recruitment of BMDM, apoptosis is reported to be found with high frequency 1 d after cranial irradiation in brain parenchyma, and diminished in a time-dependent manner by 21 d after irradiation [6]. We also examined apoptosis under our irradiation condition and got basically the same result. Although there were no cleaved caspase-3-positive cells in sections from non-irradiated MSCV-GFP mice, the cleaved caspase-3-positive cells were observed in sections from irradiated MSCV-GFP mice at 3 weeks after cranial irradiation, thereafter these cells almost disappeared at 8 weeks after cranial irradiation (data not shown). Thus, the recruitment of BMDM seems to continue after the disappearance of apoptotic cells.

Migration of BMDM may similarly be induced in human patients following brain irradiation. Whether BMDM have similar roles as resident microglia, and what the slower accumulation time-course of BMDM compared with resident microglia in the damaged areas means, are questions that remain unsolved. Given that BMDM play roles similar to those of resident microglia, BMDM could have significant therapeutic potential in neuronal tissues damaged by neurodegenerative diseases or brain tumors [1–3]. Moreover, cranial irradiation could induce other types of cells from bone marrow including stromal cells. Further studies are required to clarify mechanisms and the pathophysiological significance of BMDM and other types of bone marrow-derived cells in the irradiated brain.

## FUNDING

This work was supported by the Funding Program for Next Generation World-Leading Researchers (LS021) (to H.H.). In addition, it was supported by Grants-in-Aid from the Ministry of Education, Culture, Sports, Science and Technology of Japan for Scientific Research in Innovative Areas and from the Japan Society for the Promotion of Science for Young Scientists (to T.K.). Funding to pay the Open Access publication charges for this article was provided by Gunma University.
